# Go with the flow: a case study of migratory beekeeping and its associated costs

**DOI:** 10.1093/jee/toaf119

**Published:** 2025-06-19

**Authors:** Anja Pavlin, Andraž Marinč, Janez Prešern

**Affiliations:** Animal Production Department, Agricultural Institute of Slovenia, Ljubljana, Slovenia; Biotechnical Faculty, University of Ljubljana, Ljubljana, Slovenia; Animal Production Department, Agricultural Institute of Slovenia, Ljubljana, Slovenia; Animal Production Department, Agricultural Institute of Slovenia, Ljubljana, Slovenia

**Keywords:** honey, colonies, migration, model, CO_2_

## Abstract

The reasons for relocating honey bee colonies are often economic: to provide pollination services and/or to follow honeydew and nectar flows as they occur. In this case study, we analyzed colony migrations related to flow, modeling distances traveled, and costs per hive using official data systematically collected in Slovenia between 2014 and 2022. During this period, beekeepers recorded between 3,000 and 4,000 migrations annually. In response to economically important forage availability, between 67,000 and 96,300 hives were relocated each year. The most sought-after forage was acacia (*Robinia pseudoacacia*, 23%), followed by chestnut (*Castanea sativa*, 22%) and fir (*Abies alba*, 20%). Migration dynamics varied between years due to weather conditions, resulting in a broad range of cumulative travel distances per year, from 125,161 to 212,080 km. The estimated costs of hive transport per km included motorway tolls, fuel prices, and estimated fuel consumption. Regarding vehicle choice, using a car to relocate a small number of hives (8 to 10) was the most expensive option. A 28-hive trailer for a family car proved to be a near-optimal option, while the most cost-effective method was migration with a truck carrying 72 hives. Nonetheless, throughout the observed period, the cost of migrating 28 hives over 200 km was less than the retail value of 10 kg of honey.

## Graphical Abstract

**Graphical Abstract:**
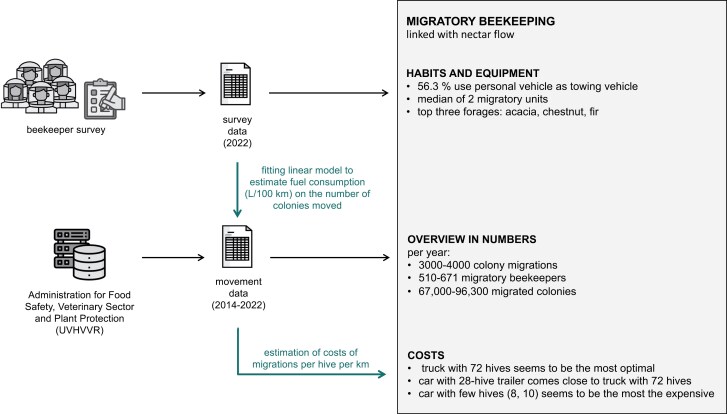

## Introduction

The main sources of revenue in apiculture are: (i) honey bee products dominated by honey but also including pollen, propolis, royal jelly, beeswax, and derived products; (ii) rearing and selling honey bee queens; (iii) rearing and selling colonies/nucs; (iv) providing pollination services; and (v) apitourism (Etxegarai-Legarreta and Sanchez-Famoso 2022). Beekeepers often focus on one of these revenue streams. From an organizational perspective, beekeepers are generally classified as either nonmigratory or migratory. In some cases, the latter are further divided into mixed (both stationary and migratory) and exclusively migratory categories (Pocol et al. 2021). While colony migration is essential in pollination services (Goodrich et al. 2019), migratory beekeeping for honey production is optional. However, relocating colonies can increase honey yield or compensate for unpredictable weather conditions (Sharma et al. 2013). It also allows beekeepers to produce different types of honey. Notably, in the EU, monofloral honeys command higher prices than blended or polyfloral honeys. For example, in Slovenia, the recommended retail price for blended floral honey was €13.3/900 g in 2023, while acacia honey was priced at €14.0/900 g, and fir honey at €17.8/900 g (Slovenian Beekeeping Association 2023; see Supplemenatary Table S1). Despite its small geographical area, Slovenia lies at the intersection of 4 biodiversity regions (Šilc et al. 2020), a factor that significantly influences the availability of resources for beekeepers. Within short distances, a wide variety of honey bee forages can be found. Several of these are large-scale and economically important, beginning with acacia flow (black locust; *Robinia pseudoacacia*) in May, followed by linden (*Tilia* spp.) in June, which often overlaps with edible chestnut (*Castanea sativa*). Among the honeydew forages, the most important is fir (*Abies alba*), typically appearing in July or August, followed by spruce (*Picea abies*), which usually appears in May (Šivic et al. 2015). However, large-scale spruce honeydew flows have become rare in recent years, with the last significant event recorded in 2017. Among the agricultural sources, 2 are particularly noteworthy: oilseed rape (*Brassica napus* subsp. *napus*) and buckwheat (*Fagopyrum esculentum*). The honey of the former is not in high demand in the local market; however, in northeastern Slovenia, it directly precedes the acacia bloom and often grows in neighboring locations. Beekeepers migrate their colonies to oilseed rape fields to support colony development before the acacia season begins.

The engine power of personal vehicles has been steadily increasing. The traditional fixed-volume cupboard-like Alberti-Žnidaršič (AŽ) hive has proven highly suitable for installation in container units or specialized trailers light enough to be towed by personal vehicles, making it easy to follow the nectar flow. However, migrating colonies comes with costs. Fuel prices, much like sugar prices, are influenced by macroeconomic and geopolitical conditions and are constantly fluctuating. Tolls are another factor that must be taken into the account when planning colony relocations.

Colony migrations also have an environmental impact. Life-cycle analysis clearly demonstrates the difference between nonmigratory and migratory beekeeping operations: 0.38 to 0.48 kg CO_2_e/kg of honey versus 1.40 to 2.20 kg CO_2_e/kg of honey (Pignagnoli et al. 2021). In addition, the biological cost of migrations must be considered. Intensive long-distance migrations shorten the lifespan of worker bees hatched immediately after relocation by 0.6 to 1.44 d, depending on the season. A similar lifespan reduction was also observed in short-distance migrations. Given that a single day represents approximately 20% of an adult worker bee’s life as a forager, this directly impacts honey yield (Simone-Finstrom et al. 2016).

In this paper, we present a case study based on population-wide data to analyze colony migrations. First, we analyzed a survey on the migratory habits and equipment of local beekeepers. Next, we examined records of colony migrations in Slovenia from 2014 to 2022, selecting those related to honey flow. Finally, we modeled travel distances and associated costs, such as fuel consumption per colony per trip and toll prices. Given the similar legislation across EU countries, this study could be expanded to encompass the entire Union.

## Materials and Methods

### Survey

We invited beekeepers to participate in the survey via social media and through an announcement in the local beekeeping magazine, providing a QR code as a direct link to the survey (Supplementary Material 1). The purpose of the survey was to collect data on (i) the mode of colony relocation and (ii) the migration-related habits and preferences of beekeepers.

To learn about the vehicles used for colony relocation, we specifically asked about the following:

- The manner of hive migration (eg migratory apiary units such as a trailers, apiary-in-container units, fixed containers on trucks, modular hives loaded in or on trucks).
- The types of vehicles used for hive migration (personal vehicle (car), truck, tractor, etc.).
- Fuel consumption during migration (liters/100 km).

Furthermore, we collected information on the following:

- The number of migratory apiary units per beekeeper.
- The number of hives per migration per unit.
- The types of forage beekeepers move their colonies to.
- The number of different forages a beekeeper attends to on a yearly basis.

### Fuel Consumption Per Colony During Migration

Survey data on fuel consumption and the number of colonies moved were used to calculate fuel costs per migration. The relationship between *fuel consumption (liters/100* *km)* and the reported *number of colonies in the first migratory unit* was determined by fitting the reported data using 2 different mathematical models (Equation (1), exponential approach; Equation (2), linear regression).


$$\begin{array}{l} \;y=A\times \left(1-e^{-\frac{x}{\tau }}\right)+B \end{array}$$ (1)

$$\begin{array}{l} y=A\;\times \;x+B \end{array}$$ (2)

To determine whether the exponential or linear approach was more appropriate, we used the Akaike information criterion (AIC), selecting the model with lower AIC score. The *lmfit* package, implementing the least-squares minimization method, was used for model fitting (Python script; available at DOI: ). The goodness of fit is reported with standard errors, also implemented within the *lmfit* package.

### Information on Beekeepers, Colonies, and Colony Relocations

Data on registered apiaries, colonies, and colony relocations were obtained from the Slovenian National Veterinary Administration (Administration for Food Safety, Veterinary Sector and Plant Protection; UVHVVR), as beekeepers are legally required to report these activities. Apiaries in the database are classified into 3 types: stationary (CBS/CBP), temporary (CBZ), and forest forage apiaries (CBG). By legal definition, a temporary (CBZ) apiary automatically expires after 30 d. If no further travel is recorded by the beekeeper, it is assumed that the colonies have been moved back to the last registered stationary apiary (CBS).

#### Beekeepers

We used a dataset on registered apiaries, which contained anonymized beekeeper unique IDs, apiary unique IDs, apiary coordinates, and colony counts within each apiary on a year-by-year basis. We excluded records with (i) missing data on colony count, (ii) missing data on unique ID, and (iii) those that reported zero colonies in every apiary under a beekeeper’s management.

#### Colony Relocations

The dataset on colony migrations contained the unique ID of the apiary of origin, unique ID of the destination apiary, destination coordinates, apiary type, number of colonies moved, and date of migration.

The 2 datasets were combined using the unique ID of the apiary of origin as a key to merge data on both origin and destination (including beekeepers’ anonymized unique IDs, unique IDs of both destination and origin apiaries, coordinates, migration dates and number of colonies moved, and apiary type at both locations). The assembled database was then checked for integrity and missing data. In the first step, we:

- Removed records with missing destination coordinates.
- Excluded origin and destination coordinates outside the boundaries of Slovenia.

In the second step, we calculated the direct distances between the origin and destination and added them to the dataset.

In the third step, we pruned/amended the data following four rules:

1. Remove migrations with a direct travel distance of less than 5 km; these were assumed to be relocations of reserve colonies.
2. Remove migrations involving fewer than 8 hives; the rationale being that the smallest apiary trailer unit is built for at least 8 hives.
3. Generate and add a return trip from temporary apiaries. As mentioned above, CBZ apiaries expire after 30 d. If a beekeeper does not provide information about further migration, it was assumed that the colonies returned to the last known stationary apiary, though this was not explicitly recorded in the raw data.
4. Remove migrations without return trips, even after applying Rule 3; if a migration ends at a stationary apiary other than the starting one, it is assumed to be a relocation for reasons other than following nectar flow (eg colony sale).

The first 2 rules represent the flow-following criteria, meaning that migrations meeting these conditions were considered migrations to forages with better nectar flow or forages unavailable at the beekeeper’s original location.

To estimate the distances traveled by beekeepers, we used the open-source version of the GraphHopper routing engine (GraphHopper GmbH, version 9.1), which was installed locally. Map data (Slovenia-latest) were downloaded from geofabrik.de on 2024-05-21. CGIAR elevation data with a resolution of 90 m were automatically obtained by editing the GraphHopper configuration file (“graph.elevation.provider: cgiar”; https://github.com/BeeKIS/honeybee_migrations). GraphHopper automatically included the available elevation data to improve travel distance accuracy. We modified the default truck routing profile by decreasing the priority of all roads except motorways (nonmotorway priority multiplied by 0.5). This adjustment ensured that the optimal routes prioritized motorways, as beekeepers would likely prefer smoother roads to minimize stress on their bees.

Each pair of coordinates (origin, destination) was queried using a Python script (https://github.com/BeeKIS/honeybee_migrations). We then processed the returned data with the Python code to obtain total travel distances as well as distances per road category.

Fuel prices were state regulated in the analyzed period throughout Slovenia, except on motorways (Supplementary Table S2). Data on fuel prices were obtained from the archive of the Ministry of the Environment, Climate and Energy and the state-owned petroleum company Petrol d.d., which operates most petrol stations in the country.

Toll data were obtained through a public information request at the communication service of DARS d.d., a government-owned company managing Slovenia’s motorways (Supplementary Table S3). Using the survey results, we assigned toll costs based on the type of towing vehicle used by most survey participants for a given number of relocated colonies.

Fuel prices and fuel consumption were then used to calculate fuel costs per hive (see above) per kilometer for each migration. Similarly, toll costs were assigned to each recorded migration.

## Results

### Survey Results

**Fig. 1.**
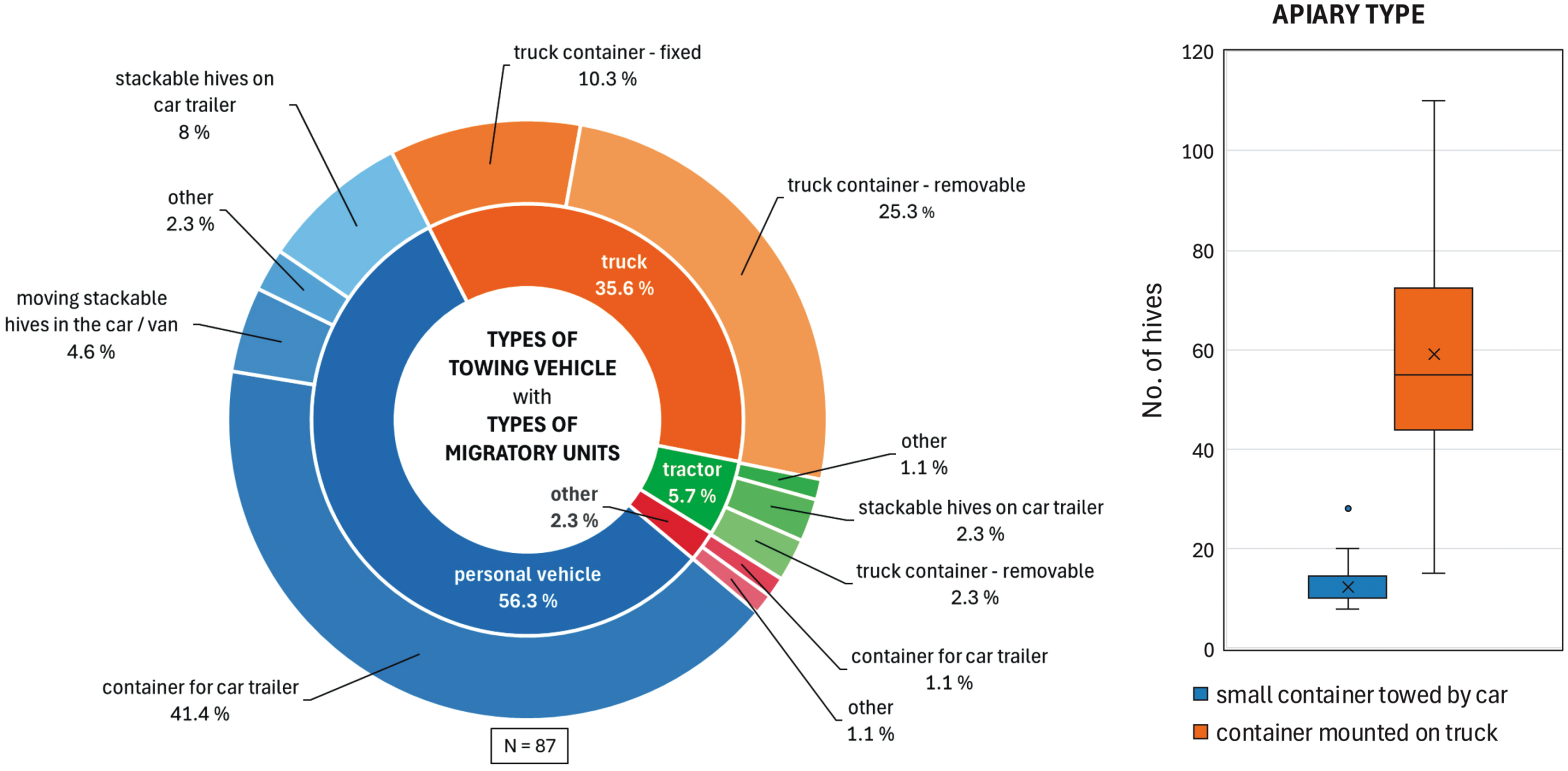
Survey results (*N* = 87). Left: types of towing vehicle used by survey participants (inner ring). Types of migratory units (apiaries) used by survey participants (outer ring). Right: number of hives (size of migratory unit) towed by personal vehicles and trucks.

We collected 87 survey responses, which, based on the pruned database (see below, Fig. 3) represent almost 14% of all beekeepers who migrated to nectar flow in 2022. A significant portion of survey participants relocate their colonies using their personal vehicles (56.3%, Fig. 1), either by towing specialized small-scale trailers for AŽ hives (41.4%) or general-use trailers loaded with box hives such as LR (8.0%). Some transport box hives inside a car or van (4.6%), while the rest did not specify the tow vehicle. The tow vehicles used by these participants are, on average, 11.3 yr old and were reported to consume between 5 and 20 liters of fuel per 100 km during migration. Another significant portion of survey participants transport their hives using an apiary-like structure, either as a fixed container-like super-structure on a truck (classified as a vehicle with a maximum allowed weight > 3.500 kg) or as a modified container loaded on a truck or truck trailer (35.6%). Additionally, 3 respondents hire a towing vehicle (truck/tractor). The corresponding towing vehicles are on average 28.7 yr old and consume between 12 and 35 liters per 100 km during migration. The number of hives relocated per trip varies; beekeepers using a car with a container mounted on a car trailer transport between 8 and 28 hives, with an average of 12 hives, while the number of hives relocated with a container mounted on a truck range between 15 and 110 (Fig. 1).

**Fig. 2.**
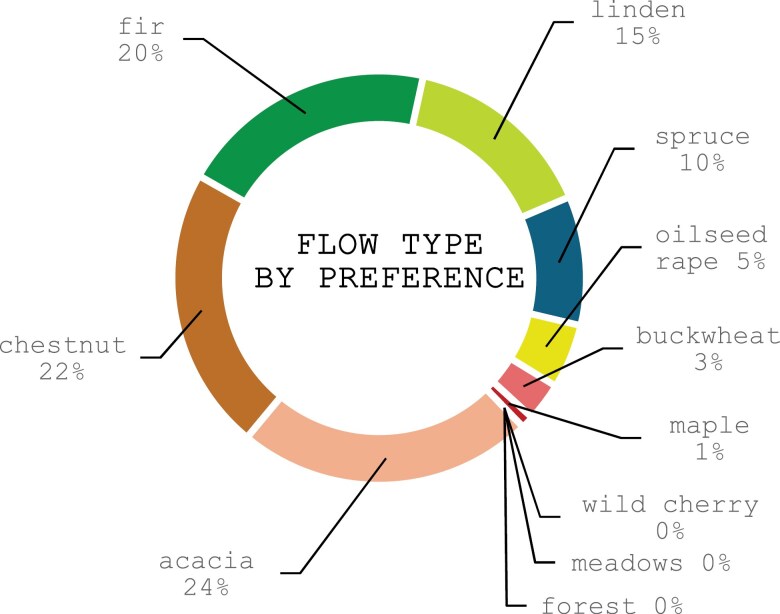
Survey results (*N* = 87). Flow type by preference of survey participants.

Survey participants own 2 migratory units (median value, range 1 to 6) and most have units of similar size (eg 10 and 12 hives). In an ideal year, participants relocate their colonies to 3 different forages (median value, with a range of 1 to 4). The most sought-after forage is acacia, accounting for 23% of migrations, followed by chestnut (22%) and fir honeydew (20%) (Fig. 2).

### Yearly Frequency and Repetition of Migrations

**Fig. 3.**
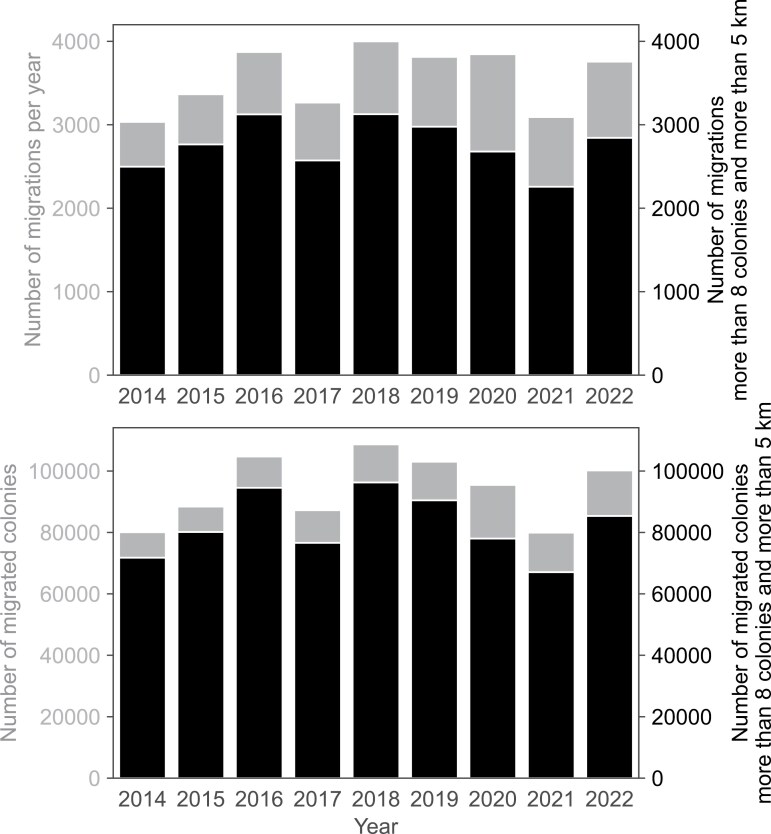
Number of registered migrations in the period 2014 to 2022 (top). Number of migrated colonies in the period 2014 to 2022 (bottom).

Between 2014 and 2022, the number of beekeepers who migrated steadily increased (Table 1). Database records also show that the number of beekeepers reporting more than zero colonies grew from 6,122 to 9,542. Beekeepers recorded between 3,000 and 4,000 migrations per year, often moving more than 100,000 colonies annually. The lowest number of migrations was recorded in 2014, which was also the year migration reporting became mandatory (Fig. 3, top; Supplementary Table S4). After pruning the dataset and removing relocations that did not meet our forage-related criteria, we estimate that between 510 and 671 beekeepers per year moved their colonies to improve honey yield, representing 6% to 9% of the total (Table 1). Among these, the small-scale beekeepers (≤28 hives) consistently accounted for 69% to 73%. These migratory beekeepers moved more than 90,000 colonies in over 3,000 migrations in 2016 and 2018, and in 2,977 migrations in 2019 (Fig. 3, bottom; Supplementary Table S4). The difference is accounted for by the beekeepers who sold colonies (single-direction relocations in the dataset), those making short distance relocations (under 5 km) likely working with reserve colonies, and those migrating fewer than 8 colonies.

**Table 1.** Active beekeepers, percentage of migratory beekeepers and percentage of small migratory beekeepers.

Year	No. of active beekeepers	No. of beekeepers relocating their colonies	No. of beekeepers relocating in relation to the flow	No. of small migratory beekeepers (≤ 28 hives)
2014	6,122	644	510 (8%)	361 (71%)
2015	7,142	672	540 (8%)	383 (71%)
2016	7,331	795	590 (8%)	426 (72%)
2017	7,979	738	569 (7%)	398 (70%)
2018	8,017	860	630 (8%)	434 (69%)
2019	8,953	849	636 (7%)	449 (71%)
2020	9,315	940	653 (7%)	475 (73%)
2021	9,542	805	576 (6 %)	404 (70%)
2022	7,603	876	671 (9%)	484 (72%)

**Fig. 4.**
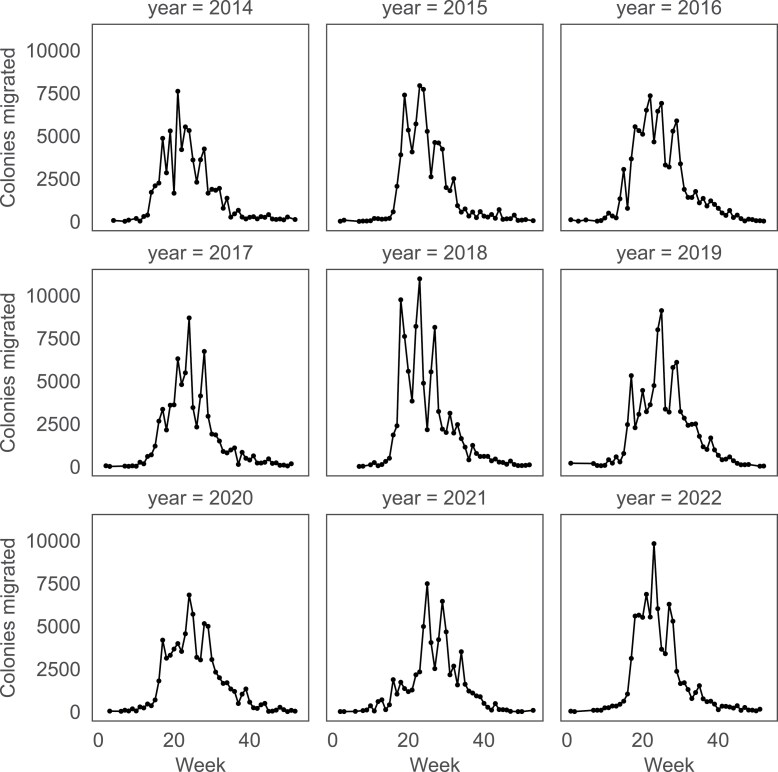
Yearly dynamics of migrations on a weekly scale. The yearly trends differ, reflecting beekeepers’ responses to weather conditions.

**Fig. 5.**
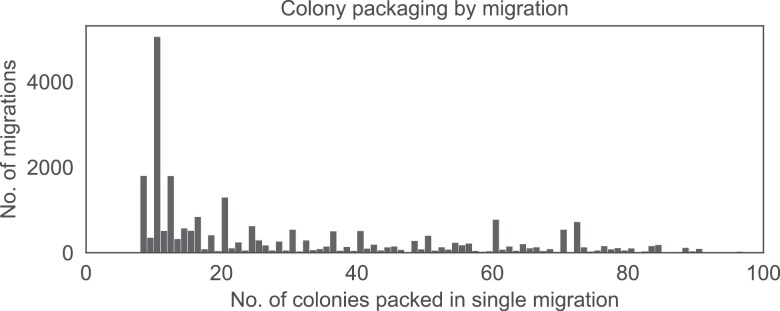
Packaging of colonies within a single relocation (2014 to 2022). Even values dominate, likely due to container design.

The yearly dynamics of migrations varied between the years. In 2018, migrations had 3 distinct peaks (Fig. 4): the first peak coincided with the acacia bloom around week 18, the second with linden and chestnut (week 23), and the third most likely with fir honeydew (week 27). In years with particularly poor spring weather, the acacia-associated peak was absent (year 2021) resulting in only 2 peaks (weeks 25 and 29). The number of migrated colonies per relocation varied (Fig. 5), depending on the mode of migration. Container units and trailers are typically symmetrically loaded, carrying an even number of colonies. The most common numbers are 8, 10, 12, 16, 18, 20, 24, and 30, with other prominent peaks at 40, 60, 70, and 72 colonies per unit. However, an add-on single-hive box is often welded onto the frame for an additional control colony equipped with a hive scale, explaining the presence of odd-numbered colony counts in the dataset. The 10-hive container is the most commonly used, followed closely by 8- and 12-hive containers.

### Analysis of Colony Migrations

**Fig. 6.**
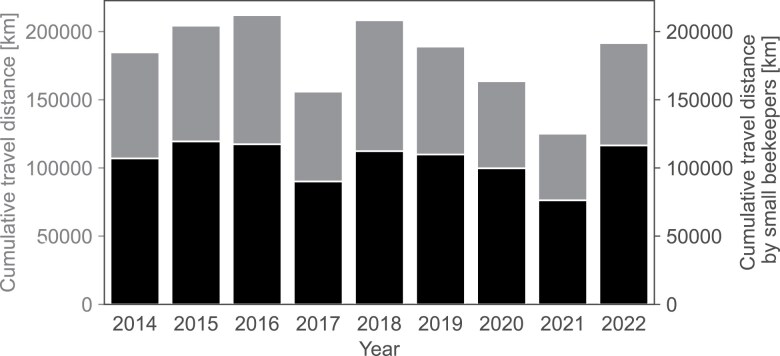
Yearly flow-related cumulative travel distances (gray). The black sections indicate distances traveled by small-scale beekeepers (with 28 or fewer colonies).

Between 2014 and 2022, there were 3 yr in which the total distance traveled for colony migrations was less than 180,000 km; all 3 yr lacked the spring peak in weekly migration dynamics (Fig. 6, c.f. Fig. 5, Supplementary Table S5). The record year was 2016, with a total of 229,478 km traveled for colony migrations (Fig. 6). Regardless of the season, between 54% (in 2018) and 61% (in 2020 to 2022) of total distance was traveled by beekeepers relocating 28 or fewer hives (Fig. 6). Travel for honey harvesting or colony inspections was not considered in this analysis.

**Fig. 7.**
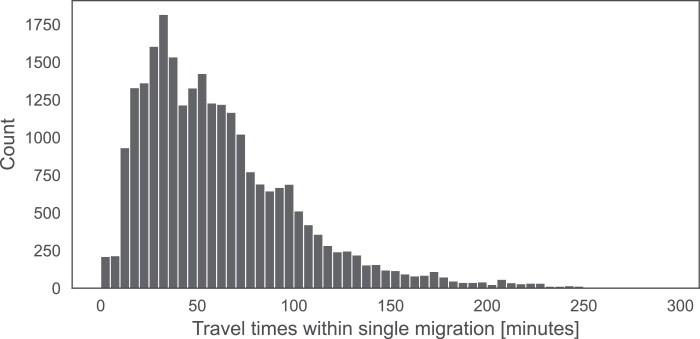
Travel time per single hive relocation in period 2014 to 2022.

**Fig. 8.**
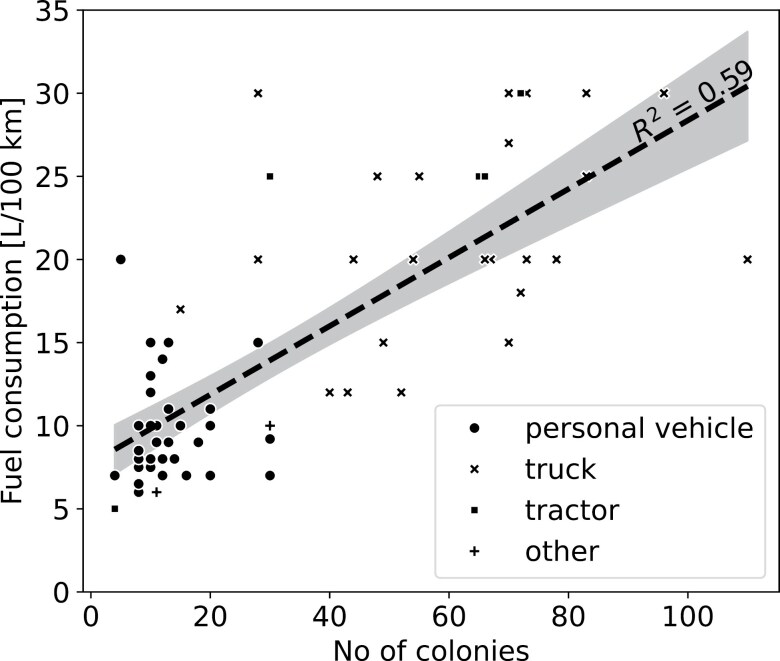
Calibration of fuel consumption as a function of the number of transported hives.

Travel time, being an important parameter for the wellbeing of the relocated colonies, was also modeled using GraphHopper. While the maximum modeled travel time was 265 min, the median travel time was 53 min (Fig. 7). The median distance traveled in a single hive relocation was 50.1 km (Q1 24.7 km; Q3 88.9 km). To describe the relationship between the number of colonies migrated and fuel consumption, we tested linear regression and rising exponential models as potential candidates. Based on the Akaike information criterion values (AIC_lin_ = −487.86 vs AIC_exp_ = −487.31), we selected the linear regression model with an intercept of 7.74, a slope of 0.206 and an *R*^2^ = 0.59, providing a robust model response for each recorded migration (Fig. 8).

**Fig. 9.**
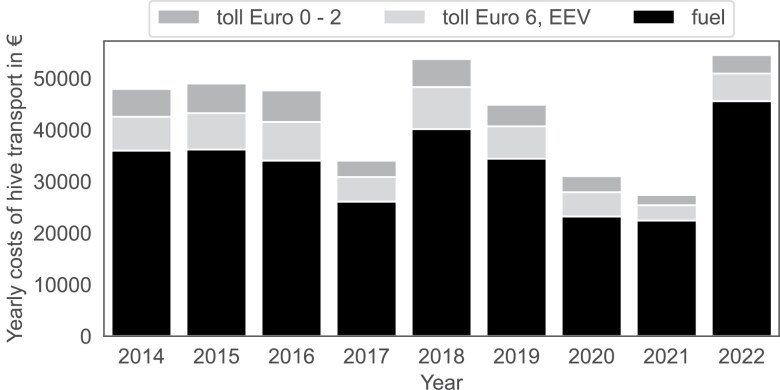
Yearly costs of colony relocations related to nectar flow. Black represents fuel costs, light gray indicates toll costs if all vehicles > 3,500 kg were Euro 6 compliant, and dark gray represents additional costs if vehicles were only Euro 0–2 compliant.

The model was then used to predict fuel consumption for each move. Combined with fuel prices on a given day, this allowed us to estimate the total fuel cost for all migrations. Motorway tolls depend on the type and emissions class of the vehicle. Since vehicle type was not recorded in the migration dataset, we estimated it based on the number of colonies recorded in each move. This estimate was informed by survey data, which indicated an overlap between the towing vehicle with the smallest truck container of 28 colonies and the largest personal car trailer with 30 hives. However, for simplicity, we made a cutoff of 28 colonies, assuming that migrations involving 28 or fewer colonies were performed using a personal vehicle and those involving more than 28 colonies with a two-axle truck. This truck belongs to vehicle category *R*^2^ and 1 of 8 emission categories (Euro 0–6, EEV). In Slovenia, annual tolls for personal vehicles cover also small trailers, meaning there are no additional costs when towing a trailer. For this reason, we did not include toll expenses in colony migration costs when personal vehicle use was presumed. We calculated the annual fuel and toll costs for colony migrations on a year-by-year basis (Fig. 9, Supplementary Table S6). The lowest total expenses for colony relocations at the national level were recorded in 2021, a year with poor spring weather, with an estimated €27,386 in the Euro 0–2 emissions scenario. The highest expenses occurred in 2022, totaling €54,511 in the most expensive Euro 0–2 scenario. On a per-colony basis, the average cost was €0.53 per colony. These estimates do not include data on fuel consumption for colony check-ups at remote locations.

### Efficiency of Hive Relocation Depends on the Number of Relocated Hives

**Fig. 10.**
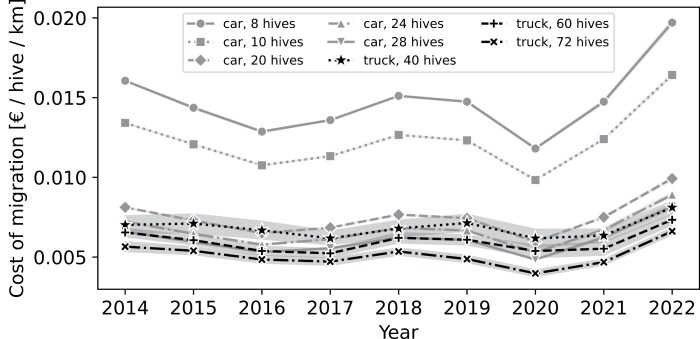
Costs of hive migrations in Euros per hive per kilometer. Comparison between towing by car and by truck. Toll costs (Euro 0–2) are included in the truck costs.

We used the modeled data to evaluate the cost per hive migration per kilometer, using 5 categories (8, 10, 20, 24, and 28 hives) of hive relocations by personal vehicle (eg car) and 3 categories relocated by truck (40, 60, and 72 hives). The costs per hive inferred from the model varied between years. The most cost-efficient method over the study period appeared to be relocating hives by truck carrying a large container apiary (≥70 hives), with costs ranging from €0.0043 ± 0.0012 per hive per km in 2020 to €0.0069 ± 0.0011 in 2022. Sixty hive units were the second most efficient option in 2017, 2021, and 2022, while 28-hive migrations by car were marginally more efficient in other years (€0.0048 ± 0.0001 in 2020). Motorway tolls accounted for approximately 20% of yearly transportation costs. Changes in toll pricing were the main reason relocations by car in some years appeared to be more efficient than those by truck. However, a car towing a container was still relatively efficient when carrying a reasonably large unit of, for instance, 24 or 28 colonies (Fig. 10). Conversely, using a car to migrate only a few hives (8, 10) was the most expensive option, with costs reaching as high as €0.01 per hive per km in 2020.

## Discussion

The analyzed survey results, combined with the veterinary administration database records and with other public data, provided unique insight into the migratory habits of beekeepers in a Central European country. The UVHVVR database is primarily designed to maintain records on apiary locations and colony counts for epidemiological purposes; consequently. it does not record the purpose of colony migrations. The criteria we established to identify migrations related to honey flow may not exclude every unrelated migration (such as hive relocation due to trade or the installation of reserve colonies in another apiary). Therefore, the presented numbers should be regarded as rough estimates. Nevertheless, the data offer a clear overview of colony migration dynamics related to nectar and honeydew flow. Differences in yearly migration patterns also clearly show that beekeepers closely follow weather conditions and other natural factors and may also consider the recommendations of the Observation and Prognostic Service (ONS), a public advisory service run by the Slovenian Beekeepers’ Association, which monitors and reports on forage flows in Slovenia.

Six hundred beekeepers following nectar flow conduct slightly fewer than 3,000 migrations annually, which aligns with survey findings indicating relocations to 3 different forage flows under ideal conditions. However, the reported number of hive relocations is likely an underestimate. We recognize that some beekeepers may avoid reporting their hive relocations out of concern that, in the event of an American foulbrood outbreak, they could face restrictions, including the immediate lockdown of all colonies within a 3 km radius. Beekeepers generally fall into 2 categories: amateur and commercial/professional. The definition of these groups typically depends on the number of hives owned; however, it is also highly dependent on regional factors, as the honeybee is widespread and beekeepers operate in varied landscapes, climates, agricultural settings and regulatory environments (Pilati and Prestamburgo 2016, Kulhanek et al. 2017). In the EU, a professional beekeeper is typically defined as someone managing at least 150 colonies (European Parliamentary Research Service 2017). The widespread use of small trailers in Slovenia suggests that most recorded relocations are carried out by small-scale beekeepers who supplement their income through honey sales. This stands in stark contrast to large-scale farming countries such as the United States and India, where pollination services are a common practice (Sharma et al. 2013, Sáez et al. 2020). In these countries, migratory beekeeping often primarily refers to pollination services, particularly in the United States, where over half of commercial honey bee colonies are used for crop pollination (USDA National Agricultural Statistics Service 2017).

The presented analysis estimates the cost of a single-direction colony migration. In a realistic scenario, where following nectar flow requires bidirectional transport, travel costs included in production pricing should naturally be doubled. For example, consider a 100 km migration of a 28-hive container by car from Central Slovenia to the Vipava Valley for the acacia flow. Including the return trip, the total travel distance is 200 km, with fuel costs amounting to €45.83. If a truck is used, an additional €38.42 must be factored in under the Euro 0–2 scheme. In comparison, studies from India and Western Australia highlight much greater migration distances. In India, single relocations range from 300 to 800 km, with 250 to 300 hives transported at once (Sharma et al. 2013, Day and White 2022). This is 5 to 6 times the median relocations in Slovenia (50 km) transporting from 8 to 110 hives.

Beyond transportation costs, apiary placement fees may also apply, particularly when migrating to forest forage apiaries (type CBG). In Slovenia, local beekeeping associations regulate forage resources, overseeing the allocation and distribution of migratory apiaries within their jurisdiction. While adherence to this system is not mandatory, and many beekeepers avoid it by making private arrangements, those who use the associations’ services must pay an apiary placement fee. The per-hive fee is set at 10% of the recommended retail price of 1 kg of honey (Magdič 2020). Unofficially, beekeepers frequently report additional “under-the-table” payments, colloquially referred to as “the sheriff’s fee”, collected by local association members in charge of assigning migratory apiary locations. This unofficial charge often doubles the cost of apiary placement. Summing all costs, the 200 km round trip hypothetical scenario would cost about €85 or €3.03 per hive. If the same trip were conducted using a 28-hive truck, with additional truck toll costs, the total expense would rise to €123.73 or €4.42 per hive (Table 2). Thus, official total costs per hive are €1.40 higher when using a truck. Additional periodic check-ups further increase costs. It is also important to consider that AŽ hives, while convenient for stacking into container units, are fixed-volume hives. During strong nectar flows, beekeepers must intervene to empty or replace full combs, increasing travel costs. Additional expenses related to hive relocation include vehicle maintenance, regular technical inspections, registration, and insurance. Larger-scale beekeepers often purchase secondhand trucks, which they modify by adding a fixed structure. The rationale behind this choice is that a more expensive or a more modern truck is unnecessary for beekeepers who have no other nonbeekeeping business operations. European environmental policies on internal combustion engines have also significantly increased travel costs for beekeepers.

**Table 2.** Colony relocation costs per transport type. Comparison between the same number of hives (28) using 2 different relocation vehicles for a 200 km round trip

	28 hive-apiary towed by car	28 hive-apiary mounted on truck
Fuel including return trip	€45.83	€45.83
Toll (Euro 0–2)	…	€38.42
Apiary placement fee (€1.41/hive in 2022)	€39.48	€39.48
**Total migration**	**€85.31**	**€123.73**

Beyond financial costs, the biological impact of colony relocations must also be considered. As noted above, long-distance transportation reduces the lifespan of honeybee workers. In a study focusing on adult bee losses, up to 45% of marked adult bees were lost due to colony relocation within 7 d after movement, while 38% drifted into neighboring colonies (Nelson and Jay 1989). Hive relocation thus affects bee orientation, although research findings on this issue remain inconsistent (Riddell Pearce et al. 2013). Colonies may also suffer from exposure to traffic pollution during migration. Diesel fumes have been shown to alter the expression of neurexin in the central nervous system of honey bees, and experimentally exposed colonies experienced greater weight loss (Reitmayer et al. 2022). Additionally, hive vibrations also increase food consumption, as shown by respirometry studies (Andress 2023). Most studies on colony relocation stress focus on long-haul migrations across North America. In contrast, Slovenia is a small country, where diverse environmental conditions can be reached in less than an hour’s drive, and the longest border-to-border motorway route can be traversed in under three and a half hours. Most hive relocations within Slovenia are short in duration, and research suggests that migrations lasting less than 1 h may not adversely affect colony foraging behavior (Riddell Pearce et al. 2013). However, colony relocations appear to be associated with lower net weight gain compared to stationary colonies in the 7 d following migration (Moeller 1975). Winter colony losses also seem to be consistently linked to migrations during the preceding season (Gray et al. 2019, 2020, 2023). Winter colony relocations (a period of low activity) increase food consumption, with colonies burning an additional 4 to 5 kg of stores per move (Moeller 1975). This increased energy expenditure may contribute to winter colony losses. However, conflicting results from various studies (Gray et al. 2019, 2020, 2023) make it difficult to explain this phenomenon.

The carbon footprint of hive relocation depends on the fuel consumed, and there appears to be a linear relationship between the 2 variables (Mickūnaitis et al. 2007). In that study, which tested new vehicles, the authors found a CO_2_ emission factor of 2.695 kg per liter of diesel. Applying this to the previously mentioned hypothetical scenario in which a container with 28 colonies undergoes a 200 km round trip with a fuel consumption of 15 liters/100 km, the total CO_2_ emissions would be 80.85 kg. Assuming an average honey yield of 10 kg per hive, or 280 kg in total, the resulting migration-related carbon footprint would be 2.89 kg of CO_2_ per kg of honey, or 0.0144 kg of CO_2_ per kg of honey per km migrated. This estimate accounts only for colony migration and excludes other emissions. Honey yield appears to be the most influential factor in such calculations. Doubling the yield halves the migration-related carbon footprint, while halving the yield doubles the migration-related carbon footprint. These values are higher than some previously reported estimates but remain within the same order of magnitude (Mujica et al. 2016, Pignagnoli et al. 2021).

The economics of honey-oriented beekeeping depend predominantly on the ratio between the price of honey multiplied by the honey yield per hive and the price of supplemental feed required per colony (eg sugar). For example, on the Slovenian domestic market, the best ratio for retail honey was achieved in 2020 (15.6:1), with a large drop in 2022 (10:1) likely driven by macroeconomic and geopolitical instability in Europe (Statistical Office of Republic of Slovenia 2024). Given these trends, we speculate that more beekeepers will adopt migratory beekeeping to maintain honey yield. The availability of hive-mounting car trailer structures, the increase in vehicle engine power and, most importantly, subsidies for mobile apiary purchases have made migratory beekeeping more accessible to small-scale producers. To our knowledge, this study represents the first large-scale analysis of migratory beekeeping practices and costs in the European Union, based on hard data. Finally, subsidized fuel for agriculture purposes (eg Croatia, Official Gazette of Republic of Croatia 2019) or central regulation of fuel prices (eg Official Gazette of Republic of Slovenia 2022) can provide relief for beekeepers, making small-scale migratory operations more viable.

## Supplementary material

Supplementary material is available at *Journal of Economic Entomology* online.

toaf119_suppl_Supplementary_Tables_S1-S6

toaf119_suppl_Supplementary_Materials
