# Supplementary material for: Go with the flow: a case study of migratory beekeeping and its associated costs
Source: J Econ Entomol. 2025 Jun 19;118(4):1485–94. doi: 10.1093/jee/toaf119 (PMC12412298; doi:10.1093/jee/toaf119)
Supplement: toaf119_suppl_Supplementary_Materials [file toaf119_suppl_supplementary_materials.docx]

**Supplemental material 1**: Survey on migratory habits and costs

1. Mode of hive transportation – type of mobile apiary unit
   1. In a container for a car trailer
   2. In a container for a truck trailer
   3. In a removable container for a truck
   4. In a fixed-mounted container on a truck
   5. In a car/van
   6. On a trailer (stackable/box hives)
   7. Other
2. How many mobile apiary units do you use?
   1. 1
   2. 2
   3. 3
   4. 4
   5. 5
   6. 6
   7. 7
   8. 8
   9. 9
   10. 10
3. How many hives do you transport on your n^th^ mobile apiary unit?

number: __

1. Type of hives in your n^th^ mobile apiary unit?
   1. AŽ
   2. AŽ 11 + 3
   3. LR
   4. other
2. Type of vehicle you use to relocate your hives?
   1. Towing with a car
   2. Towing with a tractor
   3. Loading container on a truck
   4. Truck with fixed-mounted apiary container
   5. Modified bus
   6. Other
3. Fuel consumption during hive relocation (L/100 km)?
   1. ___
4. Age of the vehicle you use to move the colonies?
   1. ___
5. How many different flows do you attempt to follow in an ideal situation?
   1. 1
   2. 2
   3. 3
   4. 4
6. Which flows do you follow?
   1. oilseed rape
   2. acacia
   3. linden
   4. edible chestnut
   5. spruce
   6. fir
   7. buckwheat
   8. other
